# Antithrombotic therapy of patients with atrial fibrillation discharged after major non-cardiac surgery. 1-year follow-up. Sub-analysis of PRAGUE 14 study

**DOI:** 10.1371/journal.pone.0177519

**Published:** 2017-05-24

**Authors:** Martina Ondrakova, Zuzana Motovska, Petr Waldauf, Jiri Knot, Lukas Havluj, Lukas Bittner, Radek Bartoska, Robert Gűrlich, Martin Krbec, Valer Dzupa, Robert Grill, Petr Widimsky

**Affiliations:** University Hospital Kralovske Vinohrady and Third Faculty of Medicine, Charles University Prague, Prague, Czech Republic; University Medical Center Freiburg, GERMANY

## Abstract

**Background:**

The study investigated the discharge antithrombotic medication in patients with atrial fibrillation (AF) after major non-cardiac surgery and the impact on one-year outcomes.

**Methods:**

A subgroup of 366 patients (mean age 75.9±10.5 years, women 42.3%, acute surgery 42.9%) undergoing major non-cardiac surgery and having any form of AF (30.6% of the total population enrolled in the PRAGUE-14 study) was followed for 1 year.

**Results:**

Antithrombotics (interrupted due to surgery) were resumed until discharge in 51.8% of patients; less frequently in men (OR 0.6 (95% CI 0.95 to 0.35); p = 0.029), and in patients undergoing elective surgery (OR 0.6 (95% CI 0.91 to 0.33); p = 0.021). Dual antiplatelet therapy was resumed more often (91.7%) in comparison to aspirin monotherapy (57.3%; p = 0.047), and vitamin K antagonist (56.3%; p = 0.042). Patients with AF had significantly higher one-year mortality (22.1%) than patients without AF (14.1%, p = 0.001). The causes of death were: ischaemic events (32.6% of deaths), bleeding events (8.1%), others (N = 51; 59.3%, 20 of them died due to cancer). Non-reinstitution of aspirin until discharge was associated with higher one-year mortality (17.6% vs. 34.8%; p = 0.018).

**Conclusion:**

Preoperatively interrupted antithrombotics were re-administrated at discharge only in half of patients with AF, less likely in male patients and those undergoing elective surgery. The presence of AF was recognized as a predictor of one-year mortality, especially if aspirin therapy was not resumed until discharge.

**Trial registration:**

ClinicalTrials.gov NCT01897220

## Introduction

Atrial fibrillation (AF) is the most common arrhythmia of adult population with the incidence of 0.4–1.0%; AF occurs in up to 5.0% of patients aged 60–70 and its prevalence significantly increases after the age of 80 [1]. The incidence of stroke in patients with non-valvular AF is between two- and sevenfold greater than that in the general population. For patients with AF caused by valvular disease, the risk of stroke is increased 17-fold [2]. However, about 15–20% of patients with AF are only on aspirin therapy (elderly patients / patients with a high risk of bleeding) [3,4].

Elderly people require surgery four times as often than the rest of the population [5]. In Europe, it is estimated that the number of patients undergoing surgery will increase by 25% by 2020. Over the same time period, the elderly population will increase by 50%. Although mortality from cardiac disease is decreasing in the general population, the prevalence of ischemic heart disease, heart failure, and cardiovascular risk factors, especially diabetes, is increasing [6]. More than 230 million major surgeries are performed annually worldwide [7]. 30-day mortality associated with moderate- to high-risk non-cardiac surgery in recent large cohorts and population-based studies exceeds 2% [8–10] and surpasses 5% in patients at high cardiac risk [11].

Non-cardiac surgery is frequently accompanied by a variable period of interrupted antithrombotic therapy, which may prevent bleeding, but it may increase the rate of cardiovascular complications. The ESC/ESA Guidelines [6]. on non-cardiac surgery recommend that vitamin K antagonist (VKA) treatment be stopped 3–5 days before surgery, and that low-molecular-weight heparin (LMWH) or unfractionated heparin (UFH) therapy be started one day after discontinuation of VKA, or later, as soon as the INR is <2.0. LMWH or UFH should be resumed at the pre-procedural dose 1–2 days after surgery, depending on the patient's haemostatic status, but at least 12 hours after the procedure. VKA should be resumed on day 1 or 2 after surgery; LMWH or UFH should be continued until the INR returns to therapeutic levels. The use of low-dose aspirin in patients undergoing non-cardiac surgery should be based on an individual decision, which depends on the perioperative bleeding risk, weighed against the risk of thrombotic complications. If it is necessary to operate on patients on dual antiplatelet therapy, the therapy should be resumed as soon as possible after surgery and, if possible, within 48 hours.

The study investigated the discharge antithrombotic medication in patients with AF after major non-cardiac surgery and the impact on one-year outcomes.

## Methods

Data for this sub-analysis were drawn from the PRAGUE-14 study (its details have already been published [12]), whose population consisted of 1200 consecutive patients (age 74.2 ±10.2 years, women 42.7%), who underwent major non-cardiac surgery (37.4% acute, 61.4% elective) in a large university hospital between 2011–2013, and who also had at least one known cardiovascular disease. The registry protocol was approved by the Ethics Committee of the University Hospital Kralovske Vinohrady in Prague (Czech Republic). Patients were included after signing an informed consent for participation. The study explored the effect of perioperative management of antithrombotic therapy on the incidence of perioperative bleeding vs. perioperative ischemic events. A sub-population of patients with AF was selected for this sub-analysis (30.6% of the whole PRAGUE-14 study population, 366 patients; age 75.9 ±10.5 years; women 42.3%; 42.9% of the surgeries were acute). Antithrombotic medication of enrolled patients was divided into three groups for the purposes of this analysis: (i) aspirin, (ii) aspirin + clopidogrel, and (iii) a VKA. We investigated whether and when the respective antithrombotic treatment was reinstituted after the major non-cardiac surgery. Patients were divided into three groups: (i) patients who left the hospital with their antithrombotic treatment reinstituted (while the treatment could be re-administered no later than at discharge), (ii) patients who restarted their antithrombotic treatment within 1 year from their hospital discharge and (iii) patients who not restarted their antithrombotic treatment within 1 year from discharge. The timing of antithrombotic treatment discontinuation and reinstitution was the responsibility of the attending surgeon in cooperation with an internal medicine specialist or a cardiologist, as appropriate. We searched for any potential predictors of early (non-)reinstitution of antithrombotic treatment at discharge. One-year outcomes were analysed. The follow up was not completed in 2.9% (N = 10) of AF patients.

### Statistical analysis

Data analysis was performed in Stata 14.1 StatSoft. Descriptive analysis was used to present continuous parameters as means ± standard deviation, while binary/categorical data were presented as counts and percentages. Binary dependent variables were analysed using univariate and multivariate logistic regression. The logistic regression analysis provided an odds ratio, the 95% confidence interval and the level of statistical significance. The level of p<0.05 was considered as statistically significant. Exact logistic regression was used if no observations were available for a combination of the binary dependent and independent variables. Logistic regression was also used to analyse 1-year outcome parameters. Kaplan-Meier plots with the log-rank test were used to visualise the one-year survival rates.

## Results

Baseline characteristics of the study population are shown in **Table 1**. AF patients were significantly older and had valve disease in their history with a higher frequency compared to non-AF patients. A part of the AF patients (N = 41) used no stable antithrombotic therapy before hospital admission; antithrombotic treatment was discontinued in all other AF patients in the perioperative period. Aspirin was discontinued in the perioperative period in 133 patients (36.3%), dual antiplatelet therapy (aspirin + clopidogrel) in 12 patients (3.3%), VKA in 177 patients (48.4%), and VKA + aspirin combination was discontinued in 3 patients. Baseline characteristics of AF patients in terms of whether they used aspirin or VKA, and whether their therapy was reinstituted until discharge is summarised in **Tables 2 and 3.** Baseline characteristics of patients with AF using dual antiplatelet therapy in S1 Table.

**Table 1:** Baseline characteristics of PRAGUE 14 study population according to presence of AF.

	*Patients with AF(n = 366)*		*Patients without AF (n = 834)*		*p (value)*
Mean age(years)	75.8 ± 9. 3		73.5 ± 10.5		<0.001
	*n*	*(%)*	*n*	*(%)*	*p (value)*
Sex (female)	155	42.3	372	31	0.438
Coronary artery disease	139	38	471	39.3	<0.001
History of myocardial infarction	65	17.8	267	22.3	<0.001
History of coronary artery bypass grafting	35	9.6	123	10.3	0.014
Valvular heart disease	71	19.4	102	8.5	0.001
Cardiomyopathy	10	37.3	13	39.5	0.181
Prosthetic heart valve	8	2.2	21	1.8	0.724
History of pulmonary embolism	8	2.2	64	5.3	0.001
History of venous thrombosis	23	6.3	60	5	0.557
History of stroke	40	10.9	87	7.3	0.812
Chronic heart failure	17	4.6	31	2.6	0.458
Diabetes mellitus	104	28.4	267	22.3	0.201
Arterial hypertension	28	78.4	644	53.7	0.725
Smoking	26	7.1	87	7.3	0.068
Chronic kidney disease	49	13.4	86	17.2	0.127
Aspirin	133	36.3	566	47.2	
Aspirin + clopidogrel	12	3.3	32	2.7	
VKA	177	48.4	117	9.8	

**Table 2:** Baseline characteristics of patients with AF according to preoperative usage of aspirin vs VKA.

	*Aspirin (n = 133)*		VKA (n = 177)		*p (value)*
Age(years)	77.5 ± 10.4		75.3 ±7.8		0.04
	*n*	*(%)*	*n*	*(%)*	
Sex (female)	61	45.9	65	36.7	0.026
Coronary artery disease	61	45.9	57	32.2	0.011
History of myocardial infarction	31	23.3	28	15.8	0.123
History of coronary artery grafting	15	11.3	15	8.5	0.396
Valvular heart disease	23	17.3	44	24.9	0.067
History of pulmonary embolism	1	0.8	6	3.4	0.163
History of venous thrombosis	6	4.5	16	9	0.111
History of stroke	18	13.5	18	10.1	0.414
Chronic heart failure	4	3	10	5.6	0.29
Diabetes mellitus	43	32.3	42	23.7	0.077
Arterial hypertension	105	78.9	144	81.4	0.562
Chronic kidney disease	22	16.5	19	10.7	0.129
Type of surgical procedure (acute)	63	47.3	64	36.2	0.039

**Table 3:** Baseline characteristics of patients with AF according to (not) re-administration of aspirin and VKA until hospital discharge.

	Aspirin re-administration		VKA re-administration	
	*until discharge/after discharge*	*p (value)*	*until discharge/after discharge*	*p (value)*
Sex (female)	n = 29/24	p = 1.000	n = 41/21	p = 0.053
Acute surgery	n = 28/29	p = 0.431	n = 34/25	p = 0.870
Elective surgery	n = 29/29		n = 59/48	
Coronary artery disease	n = 26/16	p = 0.234	n = 29/21	p = 0.865
Valvular heart disease	n = 6/8	p = 0.399	n = 20/18	p = 0.717
History of pulmonary embolism	n = 0/1	p = 0.457	n = 3/3	p = 1.000
History of venous thrombosis	n = 2/2	p = 1.000	n = 9/6	p = 0.792
History of stroke	n = 7/5	p = 1.000	n = 10/6	p = 0.792
Chronic heart failure	n = 0/3	p = 0.092	n = 8/2	p = 0.188
Diabetes mellitus	n = 16/15	p = 0.831	n = 18/23	p = 0.102

Patients with aspirin discontinued in the perioperative period (77.5±10.4 years, women 48.9%, 47.4% of them had acute surgeries) had their aspirin not re-administered until discharge in 57.2% of cases. This group of patients included 40% of patients with ischaemic heart disease and 9.1% with the history of a cerebrovascular event. Dual antiplatelet therapy (74±12 years, 68.3% women, 75.0% of them had acute surgery) was re-administered until discharge in 91.7% of cases. Patients with VKA discontinued in the perioperative period (75.3 ±7.8 years, 36.7% women, 36.2% of them had acute surgery) were discharged without any reinstitution of VKA in 43.71% of cases. This group included 14.7% patients with ischaemic heart disease, 2.7% patients with mechanical heart prosthesis and 10.7% of patients with the history of a cerebrovascular event.

AF patients were discharged with their chronic oral antithrombotic therapy in 51.8% (N = 183) of cases. 31.4% (N = 111) of patients initiated their antithrombotic therapy within one year from discharge; of these, 42 patients were discharged with bridging LMWH therapy (prophylactic dose in 41 patients; therapeutic dose in 1 patient). The initial antithrombotic therapy was not restarted during the one-year follow-up in 2.3% (N = 8) of AF patients (3 of them were discharged with a prophylactic dose of LMWH. 11.6% of patients (N = 41) were not on any antithrombotic therapy before the surgery and did not initiate any antithrombotic therapy at discharge or during the one-year follow-up, either. The reasons for resigning an antithrombotic therapy were not observed. However, it was in most cases a group of polymorbid patients with higher rates of CHADS- VASc score.

The exact time of the treatment reinstitution could not be determined in 2.9% (N = 10) of AF patients; these patients are only known to not have received any antithrombotic agents at discharge.

**Fig 1:**
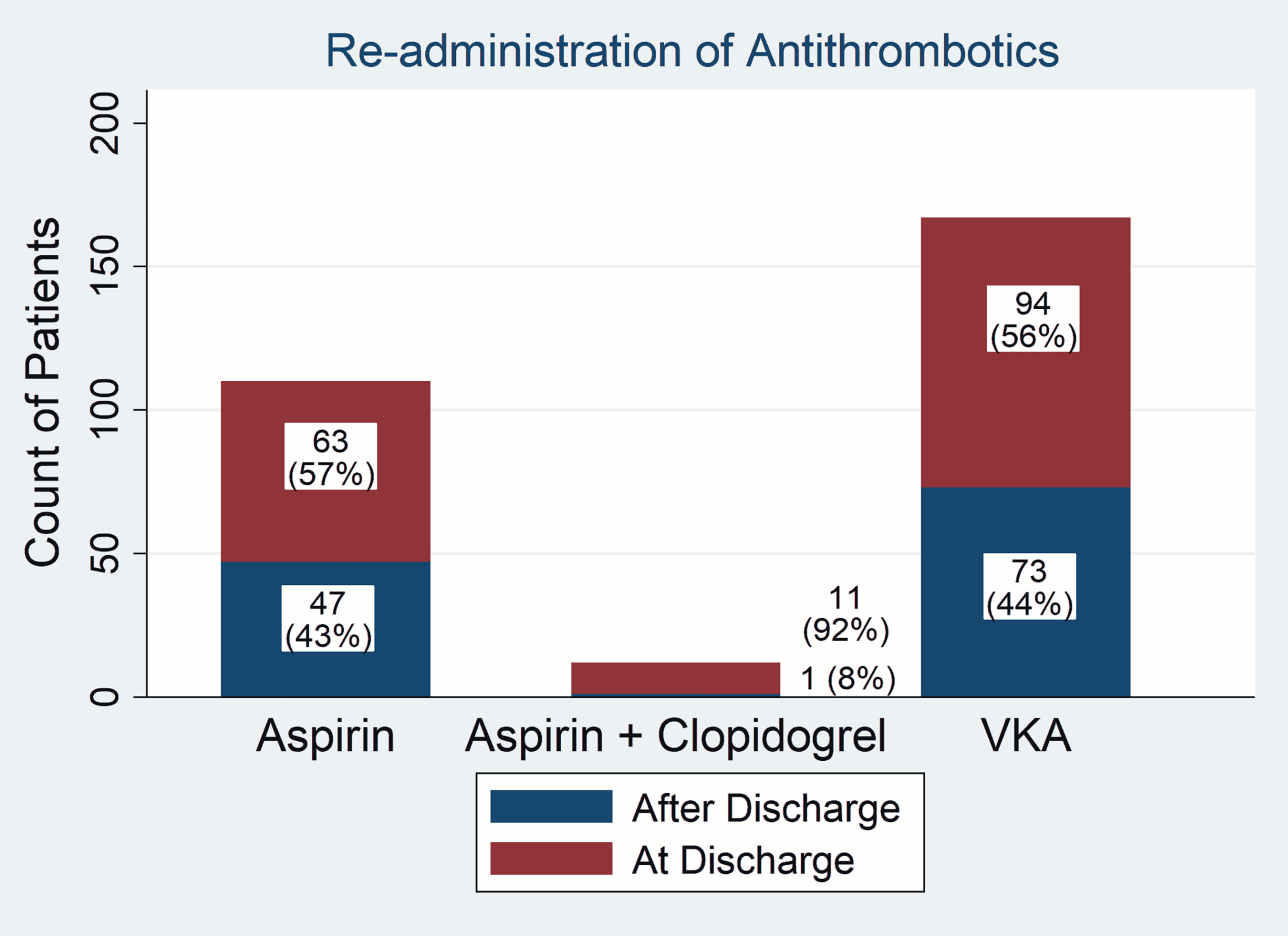
Antithrombotic treatment re-administration in the period of up to 1 year after discharge.

We found significant differences in reinstitution rates of individual medications until discharge. Dual antiplatelet therapy was reinstituted until discharge significantly more frequently (91.7%) compared to aspirin (57.3%, odds ratio 8.2, 95% CI 1.02 to 65.79; p = 0.047), and compared to VKA (56.3%, odds ratio 8.5, 95% CI 1.07 to 67.68; p = 0.042). No differences were found between VKA and aspirin reinstitution rates (p = 0.871) (**Fig 1**).

**Fig 2:**
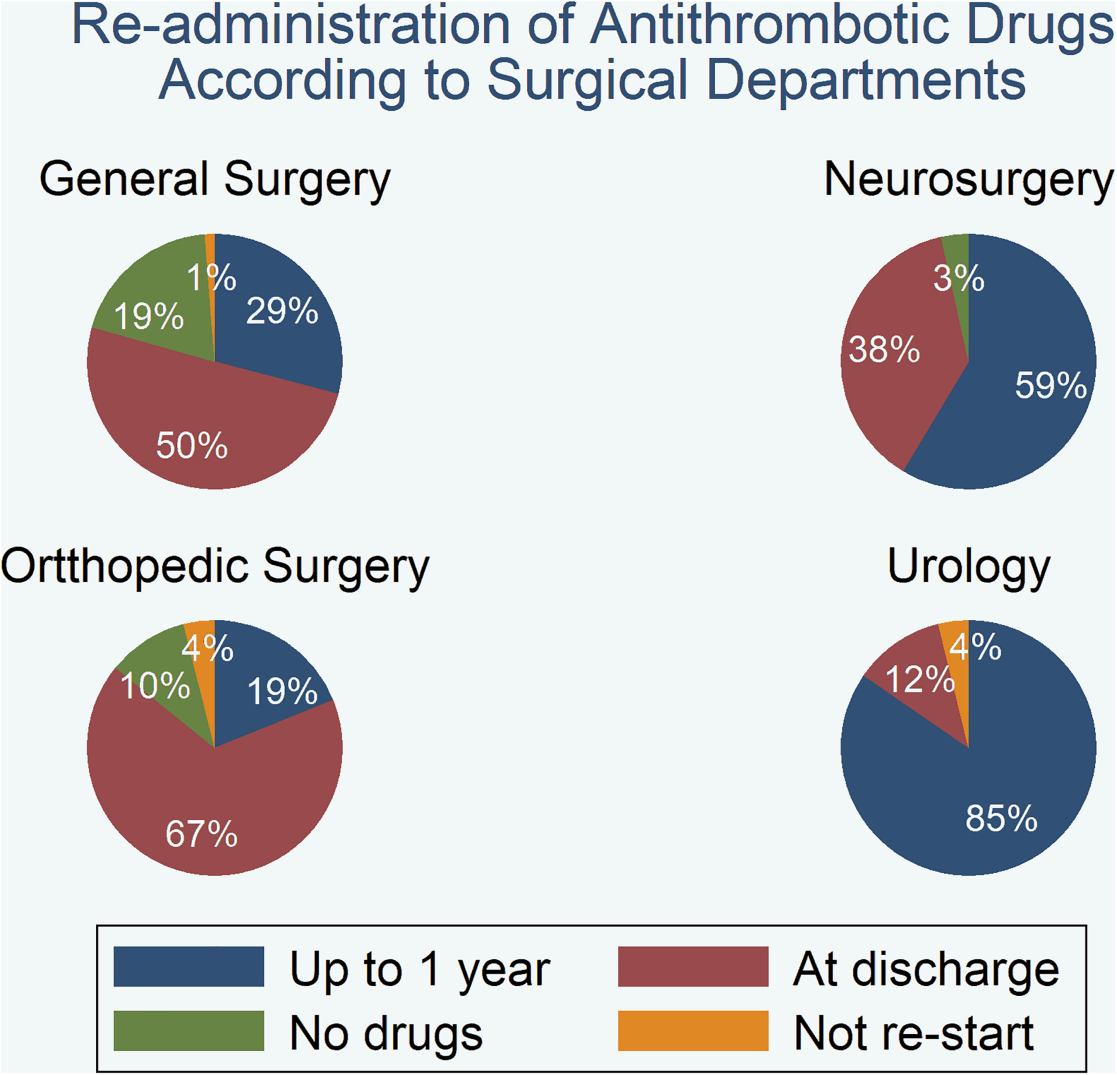
Re-administration of antithrombotic drugs according to surgery departments.

As shown by logistic regression, antithrombotic therapy reinstitution rates at discharge were significantly higher in women than men (odds ratio 1.7, 95% CI 1.05 to 2.82; p = 0.029) and in patients undergoing acute surgery compared to patients undergoing elective surgery (odds ratio 1.8, 95% CI 1.09 to 3.02; p = 0.021). We also confirmed significant differences in antithrombotic therapy reinstitution rates according to the type of surgery. Patients undergoing urological surgery had a higher probability of not re-initiating their antithrombotic treatment at discharge and; on the contrary, reinstitution of antithrombotic treatment in patients after orthopaedic surgery was significantly more likely (**Fig 2)**. The multivariate adjusted analysis confirmed the type of surgery as an independent risk factor for the re-administration of antithrombotic agents at discharge.

Perioperative ischaemic or thrombotic complications had no effect on the decision to re-administer antithrombotic therapy at discharge. Patients with perioperative ischaemic complications (n = 18) had their antithrombotic regimen reinstated until discharge in 56.1% of cases, and patients with no ischaemic complications in 63.2% (p = 0.17). Likewise, the presence of bleeding complications (n = 41) was not related to re-administration of antithrombotic treatment at discharge. Antithrombotic agents were re-administered at discharge in 56.1% of patients with bleeding complications and in 63.2% without bleeding complications (p = 0.383).

**Fig 3:**
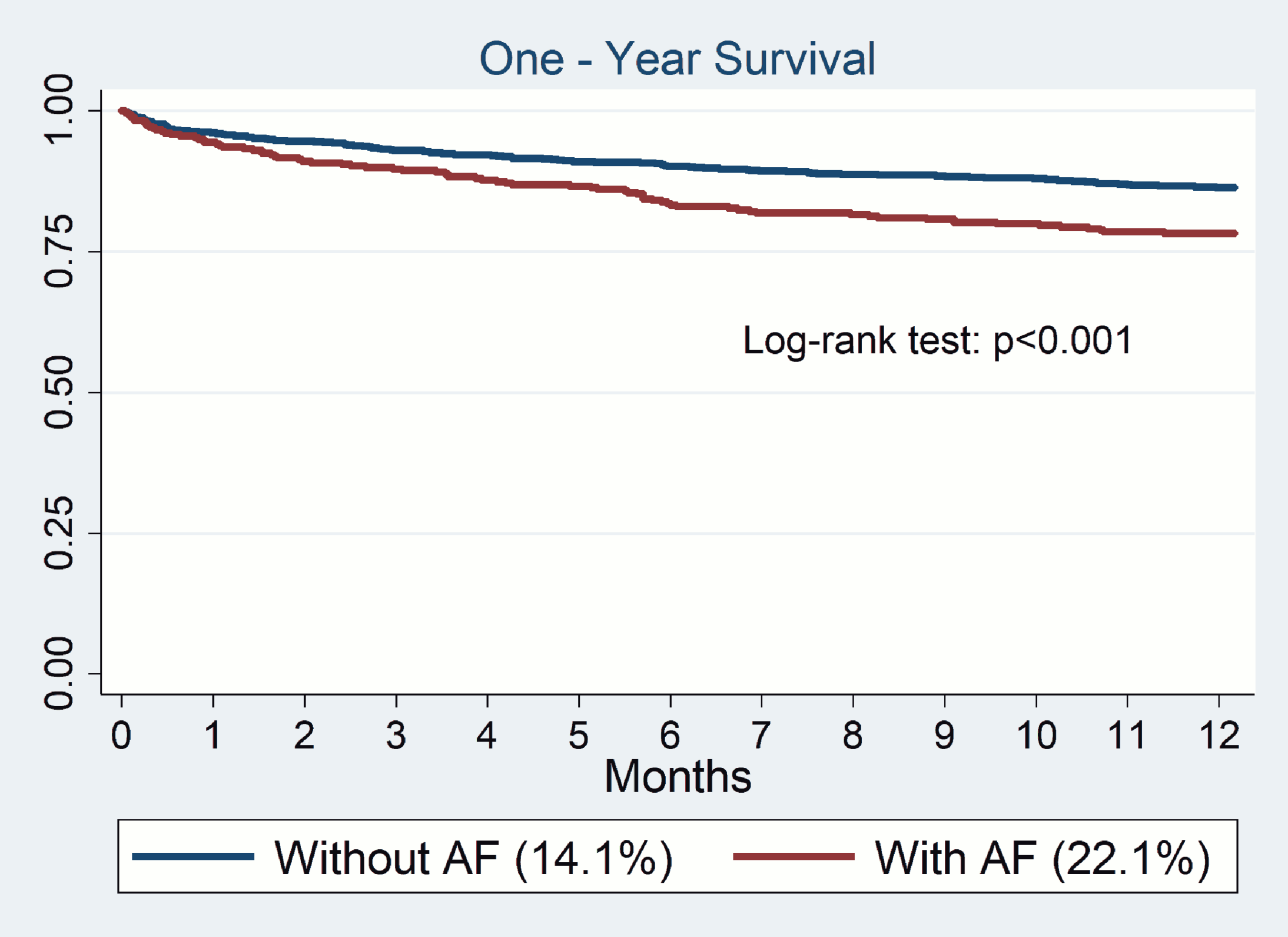
One-year survival of patients with AF after non-cardiac surgery.

Perioperative mortality in PRAGUE 14 study patients was 3.9% (N = 47), and 4.9% (N = 18) in the AF patient subpopulation. This is 4.3/5.3 -times higher than the 0.9% mortality among the remaining 17,740 patients without heart disease who underwent major non-cardiac surgery during the study period in the same institution (University Hospital Kralovske Vinohrady, Prague). AF patients showed a significantly higher one-year mortality (22.1%) compared with patients without AF (14.1%, odds 1.7, 95% Cl 1.26 to 2.37; p = 0.001) (**Fig 3)**. This difference proved to be significant even after an adjustment for age, the type of surgery, ongoing type of cancer, and chronic kidney disease with the p value after the adjustment < 0.001. The causes of mortality were: ischaemic events (N = 28; 32.6% of deaths), major bleeding events (N = 7; 8.1%), other (N = 51, 59.3%), which included 20 cancer disease (23.3% of deaths). There were observed no significant differences in one-year mortality caused by ischaemia or bleeding events associated with time of re-administration of antithrombotic drugs (until discharge x after discharge). Comparison of ischemic and bleeding events in patients without atrial fibrillation was not performed.

**Fig 4:**
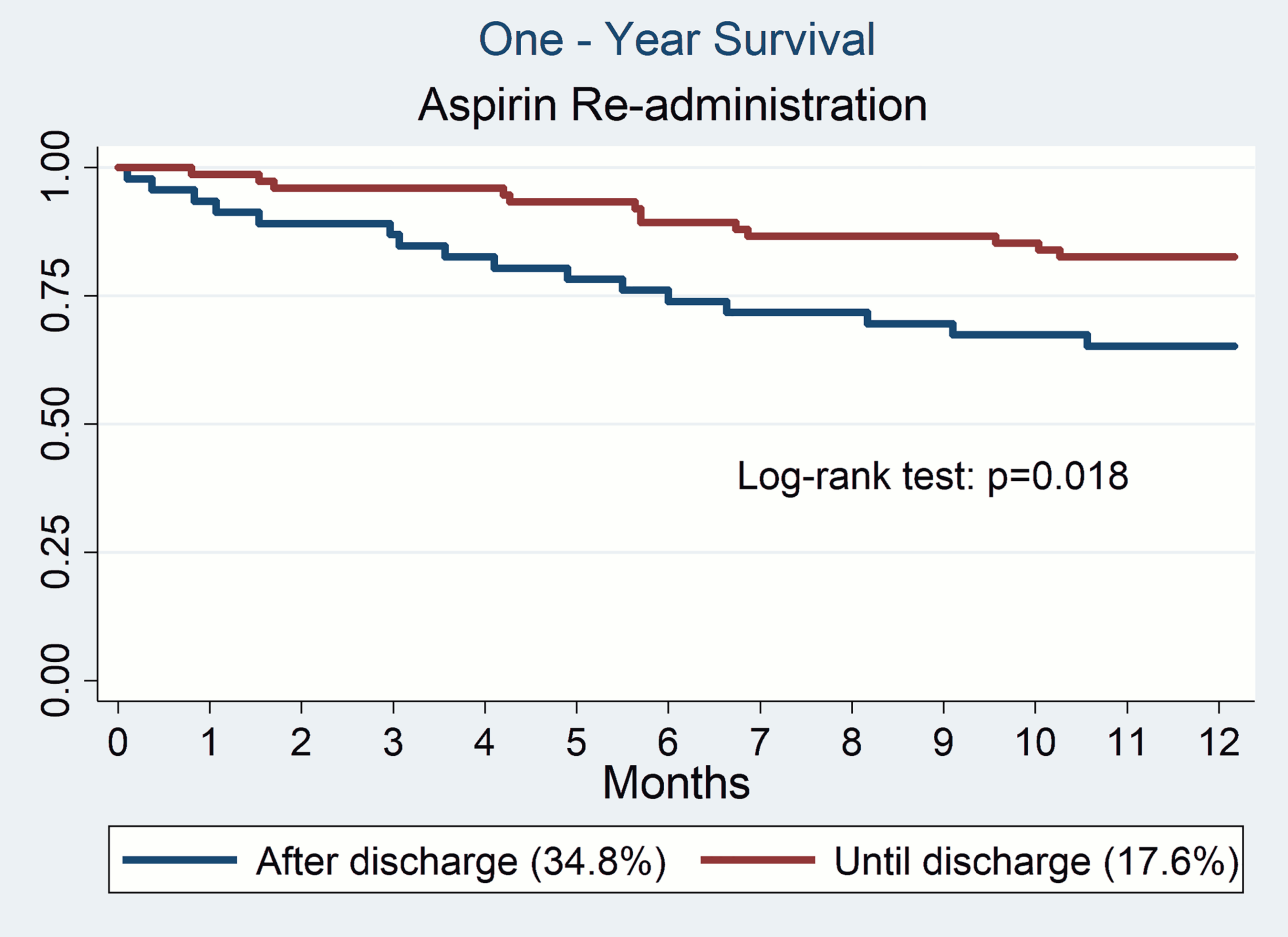
One-year survival of patients on aspirin after non-cardiac surgery.

One-year mortality in AF patients was significantly higher in the group of patients discharged after a major non-cardiac surgery without aspirin (34.8%) compared to those with their antithrombotic therapy reinstated until discharge (17.6%; p = 0.018) (**Fig 4)**. No correlation between one-year mortality and the time of re-administration of dual antiplatelet therapy (p = 0.28) or VKA (p = 0.869) was recognized.

Other one-year mortality predictors were age (OR 1.07, 95% CI 1.03–1.11; p < 0.001), perioperative ischaemic complication (OR 5.7, 95% CI 2.6–12.3; p < 0.001), chronic kidney disease (OR 2.1, 95% CI 1.08–4.3; p = 0.025), history of stroke (OR 2.1, 95% CI 1.03–4.2; p = 0.041), acute surgery (OR 3.8, 95% CI 1.98–7.06; p ≤ 0.001).

## Discussion

Cardiac patients are exposed to the risk of thromboembolism and ischaemic events in the perioperative period, while the risk of these complications persists unless their antithrombotic therapy is reinstated in the early postoperative period. The population of AF patients seems to be one of the riskiest groups in this regard given the high risk of thromboembolic events associated with this disease. There are no sufficient clinical data of patients discharged after a major non-cardiac surgery. One of the objectives of this study was to document the time of reinstatement of antithrombotic treatment in routine clinical practice in AF patients and an eventual effect on their survival. Generally, approaches to the management of antithrombotic therapy differ between surgeons and cardiologists. Surgeons prefer longer discontinuation of antithrombotic treatment due to the fear of bleeding complications, while cardiologists prefer the shortest or no treatment interruption, in a view of the increased risk of ischaemic/thromboembolic events. Antithrombotic therapy management is mostly focused on the perioperative period (during hospitalisation) while the period after the patient’s discharge is usually of lower interest. As indicated by our results, patients using dual antiplatelet therapy (undergoing acute surgery in our study population almost in all cases) were recognised by surgeons as a high-risk patient population, as documented by the 92% antithrombotic therapy reinstatement at discharge after the surgery. However, antithrombotic treatment was not reinstated at discharge in nearly one half of all the other patients (on aspirin or VKA). Higher mortality rates were found for patients not re-initiating their aspirin treatment at discharge after their surgery compared to those who did re-initiate the treatment. The incidence of perioperative complications, both in terms of bleeding or thromboembolic/ischaemic events, had no effect on reinstatement of antithrombotic therapy. Furthermore, 11% of AF patients did not use any antithrombotic medication, and another considerable proportion of the patients (36.3%) used only aspirin as their antiplatelet therapy. Higher one-year mortality after a major non-cardiac surgery in AF patients compared with patients without AF was an important finding, particularly where aspirin treatment was not re-initiated after their discharge.

Anticoagulant therapy is the most effective treatment for preventing thromboembolism in patients at high risk of stroke. The bleeding risk on aspirin is not different to the bleeding risk on VKA or novel oral anticoagulants (NOAC) but anticoagulants are superior to prevent stroke in AF patients. However, using aspirin is a reasonable option in a selected, relatively low-risk patients with high bleeding risk. The reduction in stroke risk seen with aspirin in the meta-analysis was largely driven by positive results from the SPAF1 trial, which reported an overall significant reduction in stroke risk of 42% with aspirin versus placebo [13]. Furthermore, aspirin was ineffective in stroke prevention in those over 75 years of age and did not prevent severe strokes. The only benefit shown in stroke prevention by aspirin was with a dose of 325 mg, as there seems to be no benefit by using lower doses when compared with placebo for stroke prevention in AF. The potential benefit of aspirin in the prevention of recurrent venous thromboembolism was assessed by the Warfarin and Aspirin (WARFASA) study [14] and the Aspirin to Prevent Recurrent Venous Thromboembolism (ASPIRE) study [15]. In both studies, aspirin and placebo were administered after at least 6 weeks of anticoagulation therapy in patients with venous thromboembolism. Reduced recurrence of venous thromboembolism was found in both studies, with a low risk of serious bleeding. Another study, the Aspirin for the Prevention of Recurrent Venous Thromboembolism (INSPIRE) study [16], sought to delineate the effects of the treatment in a more complex way than the previous two studies. The result was a 35% reduction in recurrent thromboembolism, which occurred in 18.4% of patients using placebo and in 13.1% of patients using aspirin (hazard ratio, 0.68; 95% CI, 0.51 to 0.90; P = 0.008).

The mortality and re-hospitalisation rates in patients with heart failure, AF and ischaemic heart disease undergoing major non-cardiac surgery were analysed in the study of Van Diepen [17]. In a group of 38,047 patients undergoing a major or minor non-cardiac surgery, this study demonstrated that patients with heart failure or AF had a significantly higher risk of death and re-hospitalisation than patients with ischaemic heart disease. Another study focused on the association of AF developed during the perioperative period (during non-cardiac vs cardiac surgery) and a long-term risk of a cerebrovascular event. Perioperative AF was shown to be associated with an increased long-term risk for ischaemic stroke especially in the case of non-cardiac surgery [18].

Preoperative risk stratification, as well as stratification before discharge in AF patients after surgery should be a stable part of care provided to patients with the history of cardiovascular disease undergoing a major non-cardiac surgery. The CHADS_2_, CHA_2_DS_2_VASc and R2CHADS2 scores used in routine clinical practice help to assess the individual risk of thromboembolism in AF patients and provide a background for the strategy and management of anticoagulation therapy in these patients. The VISION substudy [19] evaluated the risk of postoperative ischaemic stroke or death in patients with known AF undergoing other than cardiac surgery. According to the study the CHADS2 score was the best predictor of postoperative stroke/death risk in AF patients, regardless of the type of surgery.

As shown by the results of our analysis, AF in patients undergoing a non-cardiac surgery is a predictor of one-year mortality. Therefore, this group of patients should be closely monitored after their discharge from hospital after the surgery. Also, as shown by our results, cooperation between the cardiologist and the surgeon in determining a particular antithrombotic therapy for the postoperative period could actually improve the prognosis of the patients. The recommendation of the cardiologist should not be restricted as to when to discontinue the medication, but also when and under what circumstances the medication should be re-administered, or at least some bridging treatment with low-molecular-weight heparin be provided. Early postoperative antithrombotic regimen reinstatement in the absence of contraindications should be a standard procedure. Current clinical indicators (included in routinely used scoring systems) in AF patients should be taken into account when deciding on reinstatement of antithrombotic therapy in the postoperative period. As suggested by our data, aspirin therapy should be started if the patient cannot use anticoagulation therapy due to a high bleeding risk.

## Conclusion

Antithrombotic treatment discontinued in the perioperative period was re-administered at discharge only in 51.8% of AF patients, with a lower reinstatement rates in men and in patients undergoing elective surgery. Perioperative ischaemic/thromboembolic or bleeding complications had no effect on antithrombotic treatment re-initiation until discharge. The presence of AF is a predictor of one-year mortality in patients undergoing a major non-cardiac surgery, especially those without re-administration of aspirin treatment.

## Limitations

Our study has reached the main aims although there were some unavoidable limitations. The main limitation was that the presented sub-analysis was not planned and designed in parallel with the aims of PRAGUE 14 study. Consequently, the one-year follow-up data of subpopulation of patients with AF were completed 2 to 4 years after they had undergone major non-cardiac surgery.

The long interval since the date of surgery had been the main reason why we only evaluated whether the antithrombotics were re-administrated until discharge versus after discharge up to one year (and it was not possible to specify the exact time of drug reinstitution within one year). Another limitation might be, that it is difficult to compare mortality rates or the risk of thromboembolic or bleeding events in this very inhomogeneous group of patients (cancer disease was cause of death in 23.3% of patients with AF). Nevertheless, we believe that our analysis showed the subpopulation of patients with AF as a high risk and the importance of re-administration of antithrombotic medication until discharge.

## Supporting information

S1 Table Baseline characteristics of patients with AF using dual antiplatelet therapy. (DOCX)
