# Supplementary material for: Antithrombotic therapy of patients with atrial fibrillation discharged after major non-cardiac surgery. 1-year follow-up. Sub-analysis of PRAGUE 14 study
Source: PLoS One. 2017 May 24;12(5):e0177519. doi: 10.1371/journal.pone.0177519 (PMC5443499; doi:10.1371/journal.pone.0177519)
Supplement: S1 Table — (DOCX) [file pone.0177519.s001.docx]

| **Supplement Data.** Baseline characteristics of patients with AF using dual antiplatelet therapy  N= 12 (age 78±9.1) | | | | | |
| --- | --- | --- | --- | --- | --- |
| Sex (female) | n=8 |  |  |  |  |
| Acute surgery | n=9 |  |  |  |  |
| Coronary artery disease | n=10 |  |  |  |  |
| Diabetes mellitus | n= 8 |  |  |  |  |
| History of pulmonary embolism | n=0 |  |  |  |  |
| History of venous thrombosis | n=1 |  |  |  |  |
| History of stroke | n=3 |  |  |  |  |
| Until discharge x after discharge re-administration | n=11 x 1 (91.7%) |  |  |  |  |
